# Supplementary material for: Hunger- and thirst-sensing neurons modulate a neuroendocrine network to coordinate sugar and water ingestion
Source: bioRxiv. 2023 Apr 6:2023.04.06.535891. Preprint. [Version 1] doi: 10.1101/2023.04.06.535891 (PMC10104137; doi:10.1101/2023.04.06.535891)
Supplement: Supplementary file 1 [file NIHPP2023.04.06.535891v1-supplement-1.pdf]

oRxiv preprint doi: https://doi.org/10.1101/2023.04.06.535891; this version posted April 8, 2023. The copyright holder for this preprint (which was not certified by peer review) is the author/funder, who has granted bioRxiv a license to display the preprint in perpetuity. It is made available under aCC-BY 4.0 International license.

TABLES

Table S1: ISN postsynaptic partners

	Cell type / Classification	Flywire ID	Synapses from ISN 720575940619731393	Synapses from ISN 720575940628363855	Synapses from ISN 720575940625627932	Synapses from ISN 720575940624153528	Total synapses from 4 ISNs	Total synapses per cell type	Tracing contributions (number of edits)
1	FLAa2	720575940627559623	19	20	29	25	93		Jefferis Lab: Irene Salgarella (3), Varun Sane (1).
2	FLAa2	720575940613754833	19	32	21	19	91		Murthy and Seung Labs: James Hebditch (1), Ben Silverman (1), remer tancontian (1). Jefferis Lab: Bhargavi Parmar (4).
3	FLAa2	720575940621782057	18	14	21	7	60		Murthy and Seung Labs: Nash Hadjerol (6).
4	FLAa2	720575940623605180	10	10	14	14	48		Dickson Lab: Alisa Poh (1). Murthy and Seung Labs: Austin T Burke (16), Shirleyjoy Serona (1).
5	FLAa2	720575940625218590	7	16	11	10	44		Jefferis Lab: Irene Salgarella (8), Arti Yadav (1).
6	FLAa2	720575940620994292	7		23	14	44		Jefferis Lab: Irene Salgarella (1), Greg Jefferis (1), Philipp Schlegel (1), Bhargavi Parmar (1). Murthy and Seung Labs: Ben Silverman (1), Kendrick Joules Vinson (15), Joshua Bañez (2).
7	FLAa2	720575940632134483	17	6	13	6	42		Jefferis Lab: Imaan Tamimi (1), Arti Yadav (1). Murthy and Seung Labs: James Hebditch (2), Nash Hadjerol (1).
8	FLAa2	720575940619283553	11	10	9	12	42		Jefferis Lab: Arti Yadav (1). Murthy and Seung Labs: Austin T Burke (14).
9	FLAa2	720575940620306300	21		5	12	38		Murthy and Seung Labs: Austin T Burke (1), James Hebditch (2). Jefferis Lab: Arti Yadav (1).
10	FLAa2	720575940611991830	15	11	5		31		Jefferis Lab: Irene Salgarella (2). Murthy and Seung Labs: Austin T Burke (11).
11	FLAa2	720575940626512881	7		14	10	31		Murthy and Seung Labs: Mendell Lopez (10).
12	FLAa2	720575940621680264	8		10	16	34		Jefferis Lab: Anjali Pandey (1). Murthy and Seung Labs: Austin T Burke (12), J. Anthony Ocho (3).
13	FLAa2	720575940637381466	11	5		12	28		Jefferis Lab: Irene Salgarella (4). Murthy and Seung Labs: Zairene Lenizo (1).
14	FLAa2	720575940632695904	6	11	10		27		Jefferis Lab: Irene Salgarella (4), Rashmita Rana (1).
15	FLAa2	720575940631570636		6	14	6	26		Murthy and Seung Labs: Austin T Burke (5), Shirleyjoy Serona (3), Mendell Lopez (45).
16	FLAa2	720575940621605964	6		12	8	26		Seung Lab: Zhihao Zheng (1). Jefferis Lab: Irene Salgarella (9), Márcia Santos (2).
17	FLAa2	720575940637151807	8	5	13		26		Seung Lab: Zhihao Zheng (4). Jefferis Lab: A. Javier (2), Dickson Lab: Alisa Poh (3). Murthy and Seung Labs: James Hebditch (2), regine salem (1).
18	FLAa2	720575940622161032	15	9			24		Jefferis Lab: Irene Salgarella (4). Murthy and Seung Labs: Nash Hadjerol (8), Joshua Bañez (14).
19	FLAa2	720575940612087922	6		12	6	24		Jefferis Lab: Irene Salgarella (5). Murthy and Seung Labs: Austin T Burke (2), Nash Hadjerol (5).
20	FLAa2	720575940623752741	12	5	5		22		Jefferis Lab: Irene Salgarella (5).
21	FLAa2	720575940643191575		6	15		21		Murthy and Seung Labs: Mendell Lopez (11). Jefferis Lab: Arti Yadav (1).
22	FLAa2	720575940635191438		12		8	20		Jefferis Lab: Irene Salgarella (3). Murthy and Seung Labs: Zairene Lenizo (2).
23	FLAa2	720575940610624206	8		12		20		Jefferis Lab: Irene Salgarella (4). Murthy and Seung Labs: remer tancontian (2), J. Dolorosa (1).

bioRxiv preprint doi: https://doi.org/10.1101/2023.04.06.535891; this version posted April 8, 2023. The copyright holder for this preprint (which was not certified by peer review) is the author/funder, who has granted bioRxiv a license to display the preprint in perpetuity. It is made available under aCC-BY 4.0 International license.

24	FLAa2	720575940644751651	6	6	6		18		Jefferis Lab: Arti Yadav (1). Murthy and Seung Labs: Doug Bland (19).
25	FLAa2	720575940623989245		7		7	14		Jefferis Lab: Irene Salgarella (5). Murthy and Seung Labs: remer tancontian(1), Austin T Burke (1), Ben Silverman (2).
26	FLAa2	720575940623231527			9	5	14		Jefferis Lab: Yijie Yin (3). Dickson Lab: Alisa Poh (1). Murthy and Seung Labs: James Hebditch (1), Mendell Lopez (6).
27	FLAa2	720575940604764862	7		6		13		Jefferis Lab: Irene Salgarella (2). Murthy and Seung Labs: Mendell Lopez (1).
28	FLAa2	720575940628857850			13		13		Wolf Lab: fred wolf (1). Jefferis Lab: Arti Yadav (1), Yijie Yin (4). Murthy and Seung Labs: Mendell Lopez (3), remer tancontian (1).
29	FLAa2	720575940638900469	6		6		12		Jefferis Lab: Irene Salgarella (4).
30	FLAa2	720575940619398872	12				12		Jefferis Lab: A. Javier (1), Chitra Nair (1). Dickson Lab: Alisa Poh (13). Murthy and Seung Labs: Zairene Lenizo (4).
31	FLAa2	720575940631044141	6		6		12		Murthy and Seung Labs: James Hebditch (2). Jefferis Lab: Arti Yadav (1).
32	FLAa2	720575940638996413		5	7		12		Dickson Lab: Alisa Poh (8). Murthy and Seung Labs: Kyle Patrick Willie (2), Shirleyjoy Serona (3), Kendrick Joules Vinson (1), Joshua Bañez (1), James Hebditch (1). Jefferis Lab: Arti Yadav (1), Bhargavi Parmar (1).
33	FLAa2	720575940624735565	6		5		11		Dickson Lab: Alisa Poh (1). Jefferis Lab: Varun Sane (1), Arti Yadav (1). Murthy and Seung Labs: Ryan Willie (24), Rey Adrian Candilada (1).
34	FLAa2	720575940622266748		5		5	10		Jefferis Lab: Rashmita Rana (1), Zeba Vohra (1). Murthy and Seung Labs: Shirleyjoy Serona (15).
35	FLAa2	720575940644327022		5	5		10		Murthy and Seung Labs: James Hebditch (2). Jefferis Lab: Arti Yadav (1). Murthy and Seung Labs: J. Dolorosa (7).
36	FLAa2	720575940637002724	8				8		Jefferis Lab: Imaan Tamimi (1), Irene Salgarella (5). Murthy and Seung Labs: Darrel Jay Akiatan (2), J. Anthony Ocho (18).
37	FLAa2	720575940612348438				8	8		Jefferis Lab: Anjali Pandey (1). Murthy and Seung Labs: Zairene Lenizo (2), Kendrick Joules Vinson (4), Rey Adrian Candilada (3).
38	FLAa2	720575940623063847	8				8		Jefferis Lab: Arti Yadav (1). Murthy and Seung Labs: Shirleyjoy Serona (17).
39	FLAa2	720575940616872337		7			7		Jefferis Lab: Rashmita Rana (1). Murthy and Seung Labs: Ben Silverman (8), Kendrick Joules Vinson (6), Kyle Patrick Willie (1).
40	FLAa2	720575940626982165		6			6		Seung Lab: Zhihao Zheng (4). Jefferis Lab: Irene Salgarella (4), Yijie Yin (2). Murthy and Seung Labs: Nash Hadjerol (8).
41	FLAa2	720575940619403435		5			5		Jefferis Lab: Marta Costa (3), Irene Salgarella (1), Anjali Pandey (1), Varun Sane (18).
42	FLAa2	720575940626742532			5		5		Jefferis Lab: Marta Costa (3), Irene Salgarella (1), Anjali Pandey (1), Varun Sane (18).
43	FLAa2	720575940630393427			5		5		Jefferis Lab: Irene Salgarella (9).
44	FLAa2	720575940616510425		5			5		Dickson Lab: Alisa Poh (5). Murthy and Seung Labs: Rey Adrian Candilada (1).
45	FLAa2	720575940634776511			5		5		Jefferis Lab: Imaan Tamimi (2), Arti Yadav (1). Dickson Lab: Alisa Poh (2). Murthy and Seung Labs: Mendell Lopez (8), Ariel Dagohoy (19), Joshua Bañez (2), Ben Silverman (1).
46	FLAa2	720575940605589378				5	5	1080	Jefferis Lab: Arti Yadav (1). Murthy and Seung Labs: Austin T Burke (15), Zairene Lenizo (1).
47	Handshake	720575940651601910	54	51	51	35	191		Jefferis and Waddell Labs: Joseph Hsu (1). Wolf Lab: fred wolf (1). Scott Lab: Amanda Abusaif (2). Murthy and Seung Labs: Ariel Dagohoy (2), J. Anthony Ocho (2), Shirleyjoy Serona (13). Jefferis Lab: Sangeeta Sisodiya (23).
48	Handshake	720575940626449158	46	46	42	37	171		Jefferis and Waddell Labs: Joseph Hsu (1). Scott Lab: Amanda Abusaif (1).

bioRxiv preprint doi: https://doi.org/10.1101/2023.04.06.535891; this version posted April 8, 2023. The copyright holder for this preprint (which was not certified by peer review) is the author/funder, who has granted bioRxiv a license to display the preprint in perpetuity. It is made available under aCC-BY 4.0 International license.

49	Handshake	720575940618791515	45	51	49	38	183		Wolf Lab: fred wolf (2). Scott Lab: Amanda Abusaif (3).
50	Handshake	720575940622839786	39	56	44	38	177	722	Jefferis and Waddell Labs: Joseph Hsu (1). Jefferis Lab: Laia Serratosa (1), Rashmita Rana (1). Dickson Lab: Alisa Poh (1). Murthy and Seung Labs: Kyle Patrick Willie (4), remer tancontian (5), Austin T Burke (8). Itisha Joshi (15).
51	Cowboy L fragment	720575940611730674	72	95	75	59	301		Simpson Lab: Li Guo (7). Jefferis Lab: Yijie Yin (2), Siqi Fang (3). Scott Lab: Amanda González-Segarra (7), Amanda Abusaif (1). Murthy and Seung Labs: J. Anthony Ocho (2), Mendell Lopez (1), Shaina Mae Monungolh (7), Ben Silverman (1), Nash Hadjerol (1), remer tancontian (1), Miguel Albero (2), Rey Adrian Candilada (2).
52	Cowboy R	720575940624514492	36	51	38	34	159	460	Jefferis and Wilson Labs: Laia Serratosa Capdevila (6). Jefferis Lab: Yijie Yin (6), Katharina Eichler (2), Zeba Vohra (18). Scott Lab: Amanda Abusaif (6). Murthy and Seung Labs: J. Dolorosa (1), Austin T Burke (10), Darrel Jay Akiatan (13)
53	SEZ: GNG.GNG. 2607	720575940633170969	37	30	33	24	124		Murthy and Seung Labs: Ryan Willie (32), Joshua Bañez (1), remer tancontian (1), Nash Hadjerol (1). Jefferis Lab: Katharina Eichler (1), Dhwani Patel (4), Griffin Badalemente (2), Dharini Sapkal (1).
54	SEZ: PRW.GNG. 9	720575940616167730	35	19	40	21	115		Jefferis Lab: Arti Yadav (1), Griffin Badalemente (2), Dharini Sapkal (5), Yashvi Patel (2), Bhargavi Parmar (1). Scott Lab: Amanda González-Segarra (3). Murthy and Seung Lab: Mendell Lopez (12).
55	SEZ: PRW.PRW .166	720575940622732253	5	12	17		34		Jefferis Lab: Rashmita Rana (1), Zeba Vohra (4). Murthy and Seung Labs: Shirleyjoy Serona (55), Austin T Burke (1). Kim Lab: Chan Hyuk Kang (1).
56	SEZ: PRW.PRW .214	720575940625178768	8	6		17	31		Jefferis Lab: Rashmita Rana (1), Sangeeta Sisodiya (1). Murthy and Seung Labs: Ben Silverman (7), Shirleyjoy Serona (23), Austin T Burke (1), Zairene Lenizo (4), Kyle Patrick Willie (2).
57	SEZ: PRW.GNG. 6	720575940614736290	8	8	5	5	26		Murthy and Seung Labs: Ryan Willie (1), Ben Silverman (1), J. Anthony Ocho (37), Nash Hadjerol (2). Jefferis Lab: Chitra Nair (1), Yashvi Patel (1).
58	SEZ: PRW.GNG. 12	720575940619042427			8		8		Seeds Hampel Lab: Patricia Pujols (7). Murthy and Seung Labs: Zairene Lenizo (4), Rey Adrian Candilada (1), remer tancontian (1), Kendrick Joules Vinson (1). Jefferis Lab: Bhargavi Parmar (5).
59	SEZ: PRW.PRW .146	720575940621490721				6	6		Jefferis Lab: Arti Yadav (1). Murthy and Seung Labs: J. Anthony Ocho (2), Ben Silverman (3), Shaina Mae Monungolh (1), Kyle Patrick Willie (4).
60	SEZ: GNG.GNG. 1075	720575940620627803	5				5		Jefferis Lab: Shanice Bailey (3), Rashmita Rana (1), Dhwani Patel (1). Murthy and Seung Labs: J. Anthony Ocho (1).
61	SEZ: PRW.PRW .42	720575940613120721			5		5		Murthy and Seung Labs: Nash Hadjerol (9). Kim Lab: hanetwo (1).
62	SEZ: PRW.PRW .62	720575940615052812			5		5		Jefferis Lab: Arti Yadav (1). Murthy and Seung Labs: Austin T Burke (1), Zairene Lenizo (2), Rey Adrian Candilada (1), Szi-chieh Yu (1). Jefferis Lab: Dharini Sapkal (1).
63	SEZ: PRW.PRW .199	720575940624532408			5		5		Jefferis Lab: Arti Yadav (1). Murthy and Seung Labs: Jay Gager (9). Itisha Joshi (2).
64	SEZ: FLA_L.FLA _L.47	720575940631299602			5		5		Jefferis Lab: Katharina Eichler (10), Laia Serratosa (1), Marina Gkantia (11), Bhargavi Parmar (28), Dharini Sapkal (10). Murthy and Seung Labs: J. Anthony Ocho (1), Zairene Lenizo (4), Shirleyjoy Serona (41), Darrel Jay Akiatan (1). Scott Lab: Amanda Abusaif (2). Itisha Joshi (5).
65	SEZ: PRW.PRW .120	720575940619866059			5		5		Jefferis Lab: Yijie Yin (1), Rashmita Rana (1), Zeba Vohra (10). Murthy and Seung Labs: Michelle Pantujan (1), Zairene Lenizo (1), regine salem (88), Rey Adrian Candilada (1), remer tancontian (3)
66	SEZ: TRdm	720575940612921571			5		5	379	Murthy and Seung Labs: Doug Bland (5). Jefferis Lab: Imaan Tamimi (1), Laia Serratosa (1), Griffin Badalemente (1), Dhwani Patel (15).

bioRxiv preprint doi: https://doi.org/10.1101/2023.04.06.535891; this version posted April 8, 2023. The copyright holder for this preprint (which was not certified by peer review) is the author/funder, who has granted bioRxiv a license to display the preprint in perpetuity. It is made available under aCC-BY 4.0 International license.

67	DSOG 1	720575940617291323	32	33	36	22	123		Seeds Hampel Lab: Katharina Eichler (10). Jefferis and Wilson Labs: Laia Serratosa Capdevila (2). Jefferis Lab: A. Javier (8), Katharina Eichler (1), Yijie Yin (1), Dharini Sapkal (47), Arzoo Diwan (6), Dhara Kakadiya (7), Zeba Vohra (9), Dhwani Patel (10), Yashvi Patel (1). Scott Lab: Amanda Abusaif (21). Pankratz Lab: Damian Demarest (1). Murthy and Seung Labs: remer tancontian (6), J. Dolorosa (54), Kendrick Joules Vinson (3), Shaina Mae Monungolh (2), Zairene Lenizo (2). Itisha Joshi (5)
68	DSOG 1	720575940623529610	28	27	32	31	118		Seeds Hampel Lab: Katharina Eichler (3). Jefferis and Wilson Labs: Laia Serratosa Capdevila (8). Jefferis Lab: Irene Salgarella (3), Yijie Yin (2), Zeba Vohra (7), Arti Yadav (16), Bhargavi Parmar (25), Chitra Nair (39), Dhara Kakadiya (4). Scott Lab: Amanda Abusaif (12). Murthy and Seung Labs: Celia D (1), remer tancontian (2), Austin T Burke (4), Zairene Lenizo (11), Shaina Mae Monungolh (26), Rey Adrian Candilada (1), Shirleyjoy Serona (24), J. Anthony Ocho (3). Itisha Joshi (6)
69	DSOG 1	720575940623338281	19	14	8	8	49		Seeds Hampel Lab: Katharina Eichler (8). Jefferis Lab: Siqi Fang (14), Katharina Eichler (4), Irene Salgarella (2), Griffin Badalemente (7), Nidhi Patel (3), Zeba Vohra (15), Dhwani Patel (10), Chitra Nair (8), Yashvi Patel (15), Dharini Sapkal (19), Arti Yadav (4). Murthy and Seung Labs: Rey Adrian Candilada (6), Shaina Mae Monungolh (45). Kim Lab: Chan Hyuk Kang (2). Scott Lab: Amanda Abusaif (12).
70	DSOG 1	720575940644666660	13	5	17	5	40	330	Seeds Hampel Lab: Katharina Eichler, Steven Calle, Lucia Kmecova, Alexis E Santana Cruz. Murthy and Seung Labs: remer tancontian, Zairene Lenizo.
71	SEZ & SMP: FLA_L.SMP_L.27	720575940626500362	19	5	18	8	50		Jefferis Lab: Greg Jefferis (2). Dickson Lab: Alisa Poh (1). Murthy and Seung Labs: Shirleyjoy Serona (25), regine salem (17), Mendell Lopez (1), Shaina Mae Monungolh (2).
72	SEZ & SMP: VESa1	720575940632951597	12		19	16	47		Murthy and Seung Labs: Austin T Burke (72), Sarah Morejohn (1), Kyle Patrick Willie (11), James Hebditch (6), Doug Bland (5), Nash Hadjerol (39), Ben Silverman (7), J. Dolorosa (3), Zairene Lenizo (48), Mendell Lopez (9), Shaina Mae Monungolh (5), Rey Adrian Candilada (7), regine salem (80), remer tancontian (19), Darrel Jay Akiatan (13), Joshua Bañez (112), Ariel Dagohoy (9), Kendrick Joules Vinson (27), Miguel Albero (4), Shirleyjoy Serona (89), Michelle Pantujan (14), J. Anthony Ocho (11). Jefferis and Wilson Labs: Laia Serratosa Capdevila (6). Jefferis Lab: Marlon Blanquart (1), Imaan Tamimi (5), Yijie Yin (3), Irene Salgarella (3), Varun Sane (4), Griffin Badalemente (21), Philipp Schlegel (9), Dharini Sapkal (35), Chitra Nair (34), Arzoo Diwan (8), Zeba Vohra (24), Anjali Pandey (2), Dhara Kakadiya (48), Bhargavi Parmar (10), Kaushik Parmar (1), Arti Yadav (10), Yashvi Patel (12), Greg Jefferis (1). Janelia Tracers: Tansy Yang (38). Selcho Lab: Mareike Selcho (1).
73	SEZ & SMP: FLA_L.SMP_L.32	720575940627436554	12	16	6	12	46		Jefferis Lab: Greg Jefferis (1), Christophe Dunne (1), Bhargavi Parmar (1). Murthy and Seung Labs: Mendell Lopez (1), Joshua Bañez (2).
74	SEZ & SMP: FLA_R.SMP_R.32	720575940645920052	7		17	13	37		Jefferis and Wilson Labs: Laia Serratosa Capdevila (1). Wolf Lab: fred wolf (1). Huetteroth Lab: Wolf Huetteroth (1). Murthy and Seung Labs: Zairene Lenizo (6), Shirleyjoy Serona (29), Ariel Dagohoy (28), Shaina Mae Monungolh (11). Jefferis Lab: Márcia Santos (1).
75	SEZ & SMP: BiT2	720575940621662332	7	7	14	8	36		Murthy and Seung Labs: Austin T Burke (47), Joshua Bañez (9), Kyle Patrick Willie (16). Jefferis Lab: Imaan Tamimi (12), Yijie Yin (2), Bhargavi Parmar (2), Dharini Sapkal (1). Scott Lab: Amanda Abusaif (14).
76	SEZ & SMP: FLA_R.SMP_R.30	720575940637780969	5	5	10	14	34		Jefferis Lab: Arti Yadav (1), Dhwani Patel (5). Murthy and Seung Labs: Shirleyjoy Serona (3), Ryan Willie (2), Kyle Patrick Willie (2), Rey Adrian Candilada (1).
77	SEZ & SMP: Lgr3/FLAa3	720575940626452879	8	15	9		32		Jefferis Lab: Yijie Yin (1), Anjali Pandey (1). Murthy and Seung Labs: James Hebditch (1), Mendell Lopez (21), Austin T Burke (3), Joshua Bañez (3), Kendrick Joules Vinson (4).
78	SEZ & SMP: SMPpv2	720575940623701768		6	9		15		Jefferis Lab: Greg Jefferis (1), Yijie Yin (2), A. Javier (3), Irene Salgarella (5). Scott Lab: Amanda Abusaif (1). Murthy and Seung Labs: Szi-chieh Yu (15), Zairene Lenizo (9).

bioRxiv preprint doi: https://doi.org/10.1101/2023.04.06.535891; this version posted April 8, 2023. The copyright holder for this preprint (which was not certified by peer review) is the author/funder, who has granted bioRxiv a license to display the preprint in perpetuity. It is made available under aCC-BY 4.0 International license.

79	SEZ & SMP: SMPpv2	720575940628310275		7		5	12		Jefferis and Wilson Labs: Laia Serratosa Capdevila (9). Jefferis Lab: Katharina Eichler (9), Imaan Tamimi (5), Irene Salgarella (4), Philipp Schlegel (39). Murthy and Seung Labs: James Hebditch (1), Rey Adrian Candilada (10).
80	SEZ & SMP: Lgr3/FLAa 3	720575940629764906		5			5		Jefferis and Waddell Labs: Joseph Hsu (3). Murthy and Seung Labs: Austin T Burke (14). Jefferis Lab: Anjali Pandey (1).
81	SEZ & SMP: Gallinule	720575940618926757				5	5	319	Jefferis and Waddell Labs: Joseph Hsu (8). Jefferis Lab: Yijie Yin (4), Imaan Tamimi (1), Rashmita Rana (2). Murthy and Seung Labs: Mendell Lopez (2).
82	BiT	720575940610708430	71	74	93	63	301	301	Jefferis Lab: Yijie Yin (3), A. Javier (1), Imaan Tamimi (22), Varun Sane (2), Griffin Badalemente (1), Dhara Kakadiya (1), Arti Yadav (6). Murthy and Seung Labs: Claire McKellar (1), Michelle Pantujan (2), Austin T Burke (2), James Hebditch (1), J. Anthony Ocho (420), Rey Adrian Candilada (1), Nash Hadjerol (4). Wes Murfin (1). Scott Lab: Amanda Abusaif (30).
83	Ascending neuron: FLA_R.PR W.5	720575940625841241	20	6	16	27	69		Seeds Hampel Lab: Steven Calle (1). Jefferis Lab: Katharina Eichler (8). Kim Lab: Chan Hyuk Kang (2). Murthy and Seung Labs: M Sorek (2), J. Anthony Ocho (1), Zairene Lenizo (1).
84	Ascending neurons: PRW.FLA_ L.15	720575940633548128		29	33	5	67		Jefferis and Wilson Labs: Laia Serratosa Capdevila (11). Scott Lab: Zepeng Yao (1), Amanda Abusaif (2), Amanda González-Segarra (5), Rey Adrian Candilada (4).
85	Ascending neuron: FLA_L.FLA _L.25	720575940621599741	19	18		5	42		Jefferis and Waddell Labs: Joseph Hsu (3). Jefferis and Wilson Labs: Laia Serratosa Capdevila (3).
86	Ascending neuron: PRW.SMP _L.30	720575940635527092	8	5	14	11	38		Seeds Hampel Lab: Katharina Eichler (27). Jefferis and Wilson Labs: Laia Serratosa Capdevila (15). Jefferis Lab: Katharina Eichler (1), Varun Sane (1), Irene Salgarella (1), Bhargavi Parmar (10), Chitra Nair (40), Sangeeta Sisodiya (18), Zeba Vohra (4), Bhargavi Parmar (10), Dharini Sapkal (1). Murthy and Seung Labs: Zairene Lenizo (5), J. Anthony Ocho (3), Nash Hadjerol (5), Mendell Lopez (3), Joshua Bañez (4), regine salem (1), Rey Adrian Candilada (1), Kendrick Joules Vinson (3), Ariel Dagohoy (8), Kyle Patrick Willie (4), Doug Bland (31), Ryan Willie (1). Itisha Joshi (25)
87	Ascending neuron: FLA_R.PR W.2	720575940611548273	10	11	5		26		Jefferis and Wilson Labs: Laia Serratosa Capdevila (11). Murthy and Seung Labs: Mendell Lopez (1), Zairene Lenizo (2), Kyle Patrick Willie (4).
88	Ascending neuron: PRW.PRW .304	720575940631295506	7		9		16		Jefferis and Wilson Labs: Laia Serratosa Capdevila (3). Murthy and Seung Labs: Shaina Mae Monungolh (2), Nash Hadjerol (7). Jefferis Lab: Dhwani Patel (1).
89	Ascending neuron: PRW.PRW .343	720575940636263543	5		8		13		Seung Lab: Zhihao Zheng (1). Jefferis Lab: Katharina Eichler (1), Dharini Sapkal (5). Murthy and Seung Labs: Michelle Pantujan (2), Nash Hadjerol (7), Shaina Mae Monungolh (1). Dickson Lab: Alisa Poh (2).
90	Ascending neuron: PRW.PRW .137	720575940620833901			6	6	12		Murthy and Seung Labs: James Hebditch (9), Austin T Burke (11), Ben Silverman (1), Nash Hadjerol (3), Mendell Lopez (18), Ryan Willie (1), regine salem (3), Joshua Bañez (6), Rey Adrian Candilada (5), Zairene Lenizo (7), Darrel Jay Akiatan (3). Jefferis and Wilson Labs: Laia Serratosa Capdevila (3). Jefferis Lab: Katharina Eichler (10), Sangeeta Sisodiya (1), Dharini Sapkal (1), Bhargavi Parmar (37), Chitra Nair (17). Seeds Hampel Lab: Alexis E Santana Cruz (1).
91	Ascending neuron: PRW.PRW .247	720575940627304424				10	10		Jefferis Lab: Dharini Sapkal (1). Murthy and Seung Labs: Shirleyjoy Serona (16), Zairene Lenizo (1), Rey Adrian Candilada (3).
92	Ascending neuron: FLA_L.GN G.5	720575940617662950			6		6	299	Jefferis Lab: Katharina Eichler. Murthy and Seung Labs: Ben Silverman.

bioRxiv preprint doi: https://doi.org/10.1101/2023.04.06.535891; this version posted April 8, 2023. The copyright holder for this preprint (which was not certified by peer review) is the author/funder, who has granted bioRxiv a license to display the preprint in perpetuity. It is made available under aCC-BY 4.0 International license.

93	Descending neuron: PRW.GNG.16	720575940621328048	7	16	15	9	47		Jefferis and Wilson Labs: Laia Serratosa Capdevila (3). Jefferis Lab: Katharina Eichler (1), Arti Yadav (1), Dharini Sapkal (1), Dhwani Patel (1), Bhargavi Parmar (31). Murthy and Seung Labs: Austin T Burke (1), Mendell Lopez (1), Shaina Mae Monungolh (7), Ariel Dagohoy (1). Itisha Joshi (5).
94	Descending neuron: FLA_L.GNG.3	720575940610610841	9			5	14		Jefferis and Wilson Labs: Laia Serratosa Capdevila (14). Jefferis Lab: A. Javier (9), Yijie Yin (3), Katharina Eichler (1), Rashmita Rana (1).
95	Descending neuron: FLA_L.NO_OUT.7	720575940625587325	7		5		12		Jefferis Lab: Philipp Schlegel (1), Katharina Eichler (15), Paul Brooks (45). Jefferis and Wilson Labs: Laia Serratosa Capdevila (3). Selcho Lab: Mareike Selcho (7). Kim Lab: Usb (1).
96	Descending neuron: GNG.GNG.1079	720575940620668609			12		12		Seeds Hampel Lab: Katharina Eichler (2), Stefanie Hampel (1). Jefferis and Wilson Labs: Laia Serratosa Capdevila (1). Jefferis Lab: Griffin Badalemente (1), Dhwani Patel (30), Dharini Sapkal (18). Murthy and Seung Labs: regine salem (1).
97	Descending Neuron: Gumdrop	720575940630697078	5			6	11		Jefferis and Wilson Labs: Laia Serratosa Capdevila (21). Jefferis Lab: Katharina Eichler (30), Imaan Tamimi (1), Yashvi Patel (5), Arti Yadav (10), Zeba Vohra (23), Bhargavi Parmar (15). Murthy and Seung Labs: Nash Hadjerol (5). Itisha Joshi (8).
98	Descending neuron: FLA_L.NO_OUT.5	720575940619576001	6		5		11		Jefferis and Wilson Labs: Laia Serratosa Capdevila (3). Jefferis Lab: Katharina Eichler (17), Paul Brooks (28), Imaan Tamimi (1), Yijie Yin (8). Murthy and Seung Labs: Michelle Pantujan (1), J. Dolorosa (1), Ben Silverman (14).
99	Descending neuron	720575940644812398		6	5		11		Jefferis and Wilson Labs: Laia Serratosa Capdevila (4). Jefferis Lab: A. Javier (24), Katharina Eichler (4), Rashmita Rana (1), Varun Sane (1). Selcho Lab: Mareike Selcho (8). Murthy and Seung Labs: Joshua Bañez (1).
100	Descending neuron: GNG.GNG.391	720575940612692889	6				6	124	Jefferis and Wilson Labs: Laia Serratosa Capdevila (2). Jefferis Lab: Katharina Eichler (2), Zeba Vohra (1), Yashvi Patel (2). Murthy and Seung Labs: Shirleyjoy Serona (253), Darrel Jay Akiatan (5).
101	ISN	720575940625627932	5	6		5	16		Scott Lab: Alexander Edward Del Toro BSc (1). Jefferis Lab: Arti Yadav (1). Kim Lab: Hyungjun Choi (183), Chan Hyuk Kang (26), hanetwo (5). Murthy and Seung Labs: Ariel Dagohoy (2), Rey Adrian Candilada (3).
102	ISN	720575940619731393		5		5	10		Jefferis Lab: Katharina Eichler (1). Murthy and Seung Labs: Claire McKellar (1), Nash Hadjerol (2), Celia D (1), regine salem (332), Joshua Bañez (286), Kendrick Joules Vinson (8), J. Anthony Ocho (1). Scott Lab: Amanda González-Segarra (3), Alexander Edward Del Toro BSc (1). Kim Lab: Keehyun Park (6), hanetwo (19).
103	ISN	720575940628363855			5		5		Kim Group: Hyungjun Choi (29), Chan Hyuk Kang (25), hanetwo (3). Murthy and Seung Labs: Shaina Mae Monungolh (5), Joshua Bañez (1), Shirleyjoy Serona (126), Zairene Lenizo (1).
104	ISN	720575940624153528			5		5	20	Scott Lab: Amanda González-Segarra (1). Jefferis Lab: Anjali Pandey (1), Varun Sane (1). Kim Lab: Keehyun Park (1), hanetwo (22). Murthy and Seung Labs: Nash Hadjerol (12), Zairene Lenizo (4), remer tancontian (1).
								4034	

bioRxiv preprint doi: https://doi.org/10.1101/2023.04.06.535891; this version posted April 8, 2023. The copyright holder for this preprint (which was not certified by peer review) is the author/funder, who has granted bioRxiv a license to display the preprint in perpetuity. It is made available under aCC-BY 4.0 International license.

Table S2: BiT postsynaptic neurons

	Name	Neuron ID	Synapses from BiT	Total synapses from BiT per cell type	Tracing contributions (number of edits)
1	IPC	720575940650527222	54		Jefferis Lab: Imaan Tamimi (2), Anjali Pandey (1). Murthy and Seung Labs: Austin T Burke (4).
2	IPC	720575940603765280	44		Maimon Lab: Gaby Maimon (1). Jefferis Lab: Katharina Eichler (2). Murthy and Seung Labs: Austin T Burke (3).
3	IPC	720575940620932045	39		Jefferis Lab: Imaan Tamimi (2), Laia Serratosa (7). Murthy and Seung Labs: Austin T Burke (6).
4	IPC	720575940612923390	33		Jefferis Lab: Imaan Tamimi (1), Laia Serratosa (6), Markus Pleijzier (1), Rashmita Rana (1). Murthy and Seung Labs: Ryan Willie (1).
5	IPC	720575940625379859	30		Jefferis Lab: A. Javier (4), Irene Salgarella (2), Laia Serratosa (9). Murthy and Seung Labs: Austin T Burke (4).
6	IPC	720575940622897639	28		Seung Lab: Zhihao Zheng (1), Jefferis Lab: A. Javier (1). Dacks Lab: Andrew Dacks (1). Murthy and Seung Labs: Austin T Burke (7).
7	IPC	720575940643539566	26		Jefferis Lab: Imaan Tamimi (3), A. Javier (7), Laia Serratosa (4), Rashmita Rana (1). Murthy and Seung Labs: Austin T Burke (13).
8	IPC	720575940620628957	25		Jefferis Lab: Imaan Tamimi (1), A. Javier (6). Murthy and Seung Labs: Austin T Burke. (18)
9	IPC	720575940623081400	22		Jefferis Lab: Markus Pleijzier (5). Murthy and Seung Labs: Austin T Burke (18).
10	IPC	720575940631884883	22		Dacks Lab: Andrew Dacks (1). Jefferis Lab: Imaan Tamimi (1), Laia Serratosa (8). Murthy and Seung Labs: Austin T Burke (7). Itisha Joshi (2).
11	IPC	720575940628363820	22		Jefferis Lab: Imaan Tamimi (1). Dacks Lab: Andrew Dacks (1). Murthy and Seung Labs: Austin T Burke (6). Scott Lab: Meghan Laturney (1).
12	IPC	720575940624064295	22		Dacks Lab: Andrew Dacks (1). Jefferis Lab: Imaan Tamimi (3), Laia Serratosa (10), Anjali Pandey (1). Murthy and Seung Labs: Austin T Burke (5).
13	IPC	720575940628199802	21		Jefferis Lab: Markus Pleijzier (1), A. Javier (1), Laia Serratosa (9), Philipp Schlegel (1). Murthy and Seung Labs: Austin T Burke (1). Wolf Lab: Fred Wolf (1).
14	IPC	720575940623586284	17		Jefferis Lab: Imaan Tamimi (1), Márcia Santos (4). Murthy and Seung Labs: Austin T Burke (66).
15	IPC	720575940618694827	14		Jefferis Lab: A. Javier (3), Imaan Tamimi (1), Laia Serratosa (12), Anjali Pandey (1).
16	IPC	720575940614623455	10		Murthy and Seung Labs: James Hebditch (4), Nash Hadjerol (1), Doug Bland (6), Joshua Bañez (6). Jefferis Lab: Philipp Schlegel (1).
17	IPC	720575940611254681	7		Jefferis Lab: Markus Pleijzier (2), A. Javier (3), Imaan Tamimi (9). Murthy and Seung Labs: Austin T Burke (13). Kim Lab: Minsik Yun (2).
18	IPC	720575940615911380	6	442	Jefferis Lab: A. Javier (6), Laia Serratosa (6), Philipp Schlegel (1). Murthy and Seung Labs: Ryan Willie (1).
19	Lgr3/FLAa3	720575940626983185	36		Jefferis Lab: Irene Salgarella (10). Murthy and Seung Labs: James Hebditch (5), Kyle Patrick Willie (4), Rey Adrian Candilada (1), Kendrick Joules Vinson (11), Zairene Lenizo (2), Nash Hadjerol (4).
20	Lgr3/FLAa3	720575940620201084	32		Jefferis Lab: Irene Salgarella (7), Laia Serratosa (4). Murthy and Seung Labs: James Hebditch (4).
21	Lgr3/FLAa3	720575940613895934	31		Jefferis Lab: Irene Salgarella (10), Dharini Sapkal (2), Dhwani Patel (12). Murthy and Seung Labs: James Hebditch (2), Zairene Lenizo (2), Joshua Bañez (8).
22	Lgr3/FLAa3	720575940629696763	30		Jefferis Lab: Anjali Pandey (1). Murthy and Seung Labs: James Hebditch (4), Nash Hadjerol (6), Ben Silverman (1), Darrel Jay Akiatan (2).
23	Lgr3/FLAa3	720575940644053271	19		Jefferis Lab: Anjali Pandey (1), Christopher Dunne (11). Murthy and Seung Labs: Zairene Lenizo (1).
24	Lgr3/FLAa3	720575940630329903	19		Jefferis Lab: Irene Salgarella (15). Murthy and Seung Labs: James Hebditch (3).

bioRxiv preprint doi: https://doi.org/10.1101/2023.04.06.535891; this version posted April 8, 2023. The copyright holder for this preprint (which was not certified by peer review) is the author/funder, who has granted bioRxiv a license to display the preprint in perpetuity. It is made available under aCC-BY 4.0 International license.

25	Lgr3/FLAa3	720575940616987426	19		Murthy and Seung Labs: James Hebditch (4). Jefferis Lab: Anjali Pandey (8), Christopher Dunne (4), Sangeeta Sisodiya (1), Dharini Sapkal (4).
26	Lgr3/FLAa3	720575940639420032	19		Jefferis Lab: Irene Salgarella (16). Murthy and Seung Labs: James Hebditch (4), Zairene Lenizo (1).
27	Lgr3/FLAa3	720575940614714299	18		Jefferis Lab: Irene Salgarella (7). Murthy and Seung Labs: Nash Hadjerol (1).
28	Lgr3/FLAa3	720575940632879842	18		Scott Lab: Zepeng Yao (17). Jefferis Lab: Arti Yadav (1), Bhargavi Parmar (6). Murthy and Seung Labs: J. Anthony Ocho (1), Austin T Burke (1), Zairene Lenizo (2).
29	Lgr3/FLAa3	720575940626179658	15		Jefferis Lab: Irene Salgarella (10).
30	Lgr3/FLAa3	720575940626327070	15	271	Murthy and Seung Labs: Austin T Burke (1), James Hebditch (4), Ariel Dagohoy (4), Nash Hadjerol (2). Janelia tracers: Tansy Yang (2). Jefferis Lab: Irene Salgarella (8), Laia Serratosa (3). Scott Lab: Zepeng Yao (3). Pankratz Lab: Damian Demarest (2).
31	SMP & SLP: SMP_L.SMP_L.276	720575940617650203	45		Jefferis and Waddell Labs: Joseph Hsu (2). Murthy and Seung Labs: Kyle Patrick Willie (32). Jefferis Lab: Laia Serratosa (1), Anjali Pandey (1), Yijie Yin (4).
32	SMP & SLP: SMP_R.SMP_R.278	720575940619352198	44		Murthy Lab: Lucas Encarnacion-Rivera (1), Bock Lab: Davi Bock (4). Jefferis Lab: Yijie Yin (13), Greg Jefferis (15).
33	SMP & SLP: SMPpv1; right	720575940631373869	38		Jefferis and Waddell Labs: Joseph Hsu (2). Jefferis Lab: Irene Salgarella (1). Murthy and Seung Labs: J. Dolorosa (2), Kyle Patrick Willie (4).
34	SMP & SLP: SMPpv1; left	720575940633754292	26		Jefferis Lab: Yijie Yin (2). Murthy and Seung Labs: Ben Silverman (1).
35	SMP & SLP: SMP_R.SMP_R.808	720575940635354981	12		Murthy and Seung Labs: Zairene Lenizo (1), Nash Hadjerol (24), remer tancontian (5). Jefferis Lab: Anjali Pandey (1).
36	SMP & SLP: SMPpd1; left	720575940627327750	12		Jefferis Lab: Rashmita Rana (1). Murthy and Seung Labs: James Hebditch (3), remer tancontian (1).
37	SMP & SLP: SMP_R.SMP_R.70	720575940611332722	11		Jefferis Lab: Yijie Yin (1), Rashmita Rana (1). Murthy and Seung Labs: Kyle Patrick Willie (10.
38	SMP & SLP: SMPpd1; left	720575940619099558	10		Jefferis Lab: A. Javier (2), Arti Yadav (1).
39	SMP & SLP: SMP_R.SMP_R.742	720575940631589407	9		Murthy and Seung Labs: Austin T Burke (17), James Hebditch (2), Kendrick Joules Vinson (1). Jefferis Lab: Anjali Pandey (1).
40	SMP & SLP: SLPal1	720575940618140283	8		Jefferis Lab: A. Javier (3). Murthy and Seung Labs: Nash Hadjerol (1), Ariel Dagohoy (1), James Hebditch (2).
41	SMP & SLP: SMPpd2	720575940619949556	7		Jefferis Lab: Irene Salgarella (3), Anjali Pandey (1).
42	SMP & SLP: SMP_R.SMP_R.757	720575940632526547	6		Murthy and Seung Labs: Austin T Burke (1), Kyle Patrick Willie (6), Mendell Lopez (10), Doug Bland (24), remer tancontian (6). Jefferis Lab: Anjali Pandey (1).
43	SMP & SLP: SMP_L.SMP_L.706	720575940629298679	6		Murthy Lab: Lucas Encarnacion-Rivera (3). Jefferis Lab: A. Javier (1), Varun Sane (8), Arti Yadav (1). Murthy and Seung Labs: Kendrick Joules Vinson (7), remer tancontian (2).
44	SMP & SLP: SMP_R.SMP_R.906	720575940640456923	6		Jefferis Lab: Philipp Schlegel (3), Yijie Yin (1), Varun Sane (6), Anjali Pandey (1). Selcho Lab: Mareike Selcho (17). Murthy and Seung Labs: Mendell Lopez (19), Kyle Patrick Willie (1).
45	SMP & SLP: DM3; right	720575940621647498	5		Jefferis Lab: A. Javier (1), Yijie Yin (1).
46	SMP & SLP: DM3_canonical	720575940619899668	5		Jefferis Lab: Yijie Yin (16). Murthy and Seung Labs: Nash Hadjerol (1), Kyle Patrick Willie (1).
47	SMP & SLP: SMP_R.SMP_R.677	720575940629901307	5		Jefferis Lab: Yijie Yin (2), Chitra Nair (1), Varun Sane (3). Murthy and Seung Labs: Zairene Lenizo (2).
48	SMP & SLP: SLP_L.LH_L.5	720575940616707545	5		Jefferis and Wilson Labs: Laia Serratosa Capdevila (1). Wes Murfin (4). Jefferis Lab: Yijie Yin (3), Varun Sane (1), Dhara Kakadiya (2), Chitra Nair (4). Murthy and Seung Labs: Doug Bland (3), Kendrick Joules Vinson (23), Joshua Bañez (10), Rey Adrian Candilada (1).
49	SMP & SLP: SLP_R.SLP_R.557	720575940629163419	5		Murthy and Seung Labs: Austin T Burke (3). Jefferis Lab: Tomke S (14), Griffin Badalemente (4), Yijie Yin (1), Varun Sane (4), Dhwani Patel (1).

bioRxiv preprint doi: https://doi.org/10.1101/2023.04.06.535891; this version posted April 8, 2023. The copyright holder for this preprint (which was not certified by peer review) is the author/funder, who has granted bioRxiv a license to display the preprint in perpetuity. It is made available under aCC-BY 4.0 International license.

50	SMP & SLP: SMP_L.SMP_L.578	720575940626063688	5	270	Murthy and Seung Labs: Austin T Burke (13).
51	CCHa2R (RA)	720575940638190133	65		Murthy and Seung Labs: Claire McKellar (1). Jefferis Lab: Anjali Pandey (1), Laia Serratosa (117), Dhwani Patel (3). Scott Lab: Amanda Abusaif (1), Zepeng Yao (27). Kim Lab: hanetwo (1).
52	CCHa2R (RA)	720575940615181910	64		Jefferis Lab: Anjali Pandey (1). Scott Lab: Amanda Abusaif (12). Murthy and Seung Labs: Michelle Pantujan (41), Ryan Willie (459).
53	CCHa2R (RA)	720575940629642460	50		Jefferis and Waddell Labs: Joseph Hsu (1). Murthy and Seung Labs: Claire McKellar (12), Ryan Willie (1), Shirleyjoy Serona (1), Doug Bland (296), J. Anthony Ocho (3). Jefferis Lab: Arti Yadav (1). Kim Lab: Hyungjun Choi (74).
54	CCHa2R (RA)	720575940611411162	49	228	Murthy and Seung Labs: Claire McKellar (2), Nash Hadjerol (2), Zairene Lenizo (1). Jefferis Lab: Irene Salgarella (3). Scott Lab: Amanda Abusaif (11). Kim Lab: hanetwo (14),Chan Hyuk Kang (2).
55	SMP & SEZ: ADM09	720575940628462927	48		Anderson Lab: Altyn Rymbek (2). Jefferis Lab: Imaan Tamimi (6), Laia Serratosa (10), Rashmita Rana (1), Sangeeta Sisodiya (1). Murthy and Seung Labs: James Hebditch (1), Joshua Bañez (1), Mendell Lopez (58), Zairene Lenizo (2), J. Dolorosa (1). Kim Lab: Minsik Yun (1).
56	SMP & SEZ: pCd1?	720575940618057095	22		Jefferis Lab: A. Javier (1), Irene Salgarella (1). Murthy and Seung Labs: Austin T Burke (23)
57	SMP & SEZ: pCd1?	720575940604332460	12		Seung Lab: Zhihao Zheng (1). Jefferis and Wilson Labs: Laia Serratosa Capdevila (1). Jefferis: A. Javier (1), Bhargavi Parmar (1). Murthy and Seung Labs: Austin T Burke (13).
58	SMP & SEZ: ADM09p	720575940642910152	12		Scott Lab: Zepeng Yao (6). Murthy and Seung Labs: Austin T Burke (3), Shaina Mae Monungolh (1). Jefferis Lab: Yijie Yin (4), Marina Gkantia (10).
59	SMP & SEZ: DM3	720575940626005330	11		Janelia tracers: Tansy Yang (5). Jefferis Lab: Yijie Yin (3). Murthy and Seung Labs: Austin T Burke (7).
60	SMP & SEZ: Dh44	720575940618579505	10		Jefferis Lab: Imaan Tamimi (1), A. Javier (4), Rashmita Rana (1). Murthy and Seung Labs: Austin T Burke (13), remer tancontian (7).
61	SMP & SEZ: pCd1?	720575940645882420	10		Jefferis and Waddell Labs: Joseph Hsu (1). Murthy and Seung Labs: Austin T Burke (18), Joshua Bañez (1).
62	SMP & SEZ: pCd1?	720575940638671219	10		Jefferis Lab: A. Javier (3), Irene Salgarella (2), Tomke S (2), Varun Sane (5). Murthy and Seung Labs: Claire McKellar (2), remer tancontian (5), Nash Hadjerol (7).
63	SMP & SEZ: pCd1?	720575940622119861	8		Jefferis Lab: Irene Salgarella (2), Varun Sane (5), Yijie Yin (2). Murthy and Seung Labs: Austin T Burke (21).
64	SMP & SEZ: DM2_dorsal	720575940629754588	8		Anderson Lab: Altyn Rymbek (1). Jefferis Lab: Yijie Yin (11), Varun Sane (4). Murthy and Seung Labs: Austin T Burke (1), Joshua Bañez (1).
65	SMP & SEZ: SMPpv2	720575940621362044	7		Jefferis and Wilson Labs: Laia Serratosa Capdevila (1). Jefferis Lab: Yijie Yin (34), Irene Salgarella (1), Imaan Tamimi (7), Chitra Nair (1). Murthy and Seung Labs: Shirleyjoy Serona (1), Austin T Burke (1).
66	SMP & SEZ: FLA_R.NO_OUT.6	720575940623184567	7		Jefferis Lab: Katharina Eichler (1), Philipp Schlegel (1). Murthy and Seung Labs: Austin T Burke (1), Ben Silverman (2).
67	SMP & SEZ: pCd1?	720575940638719907	7		Jefferis and Waddell Labs: Joseph Hsu (1). Murthy and Seung Labs: Austin T Burke (1), Kyle Patrick Willie (6). Wes Murfin (29).
68	SMP & SEZ: pCd1?	720575940612572054	6		Seung Lab: Zhihao Zheng (1). Jefferis Lab: Irene Salgarella (6). Murthy and Seung Labs: Austin T Burke (37).
69	SMP & SEZ: DM2	720575940608174894	5		Anderson Lab: Altyn Rymbek (1), Jefferis and Waddell Labs: Joseph Hsu (14).
70	SMP & SEZ: DM2	720575940635196334	5		Jefferis Lab: Irene Salgarella (6). Janelia tracers: Tansy Yang (11). Murthy and Seung Labs: Austin T Burke (7).
71	SMP & SEZ: pMP5/DM2	720575940628732610	5		Murthy and Seung Labs: Austin T Burke (4), remer tancontian (5). Jefferis Lab: Irene Salgarella (3), Yijie Yin (22). Janelia tracers: Tansy Yang (2).
72	SMP & SEZ: SMP_R.FLA_L.29	720575940636113264	5		Jefferis Lab: Greg Jefferis (1), Laia Serratosa (11), Anjali Pandey (1). Dickson Lab: Alisa Poh (1). Murthy and Seung Labs: Nash Hadjerol (11).
73	SMP & SEZ: pCd1?	720575940638285184	5	203	Murthy and Seung Labs: Austin T Burke (3). Wes Murfin (9). Jefferis Lab: Laia Serratosa (4), Varun Sane (4).
74	Antler L	720575940622998967	54		Murthy and Seung Labs: Austin T Burke (17), Zairene Lenizo (4), Darrel Jay Akiatan (1), Shirleyjoy Serona (12), Shaina Mae Monungolh (2). Jefferis Lab: Yijie Yin (3).

bioRxiv preprint doi: https://doi.org/10.1101/2023.04.06.535891; this version posted April 8, 2023. The copyright holder for this preprint (which was not certified by peer review) is the author/funder, who has granted bioRxiv a license to display the preprint in perpetuity. It is made available under aCC-BY 4.0 International license.

75	Antler R	720575940631226439	50	104	Murthy and Seung Labs: Austin T Burke (17), Rey Adrian Candilada (3). Jefferis Lab: Varun Sane (7), Yijie Yin (5).
76	visual projection: SMP_R.SMP_R.201	720575940616608837	23		Jefferis Lab: Irene Salgarella (1), Rashmita Rana (1), Griffin Badalemente (4). Murthy and Seung Labs: J. Anthony Ocho (12), J. Dolorosa (1).
77	visual projection: SMP_R.SMP_R.698	720575940630512711	17		Jefferis Lab: Arti Yadav (1). Murthy and Seung Labs: Doug Bland (19).
78	visual projection: SMP_L.SMP_L.880	720575940636320063	15		Murthy and Seung Labs: J. Dolorosa (26). Jefferis Lab: Rashmita Rana (1).
79	visual projection: SMP_L.SMP_L.362	720575940620022960	12	67	Kim Lab: Dustin Garner (2). Jefferis Lab: Yijie Yin (16). Murthy and Seung Labs: Ben Silverman (5), remer tancontian (1).
80	Fan shaped body: DL1_dorsal; left	720575940626612254	29		Jefferis Lab: Rashmita Rana (1), Zeba Vohra (1). Murthy and Seung Labs: Austin T Burke (3), Kyle Patrick Willie (1).
81	Fan shaped body: FBl1-2	720575940642476448	17		Murthy and Seung Labs: Jay Gager (1), James Hebditch (4), Kendrick Joules Vinson (1). Jefferis Lab: Varun Sane (6), Yijie Yin (3).
82	Fan shaped body: FB.FB.473	720575940619615936	7		Jefferis Lab: Imaan Tamimi (1), A. Javier (1), Laia Serratosa (3), Arti Yadav (1), Sangeeta Sisodiya (7).
83	Fan shaped body: DL1	720575940613052200	5	58	Jefferis Lab: Varun Sane (3), Griffin Badalemente (1). Murthy and Seung Labs: Shaina Mae Monungolh (1).
84	Gallinule	720575940617054621	21		Jefferis Lab: Anjali Pandey (1). Murthy and Seung Labs: Mendell Lopez (1), Joshua Bañez (2).
85	Gallinule	720575940630349905	18		Seeds Hampel Lab: Lucia Kmecova (1). Huetteroth Lab: Wolf Huetteroth (3). Jefferis Lab: Laia Serratosa (3).
86	Gallinule	720575940629913130	7		Murthy and Seung Labs: Zairene Lenizo (12).
87	Gallinule	720575940620228449	5		Jefferis Lab: Yijie Yin (1). Murthy and Seung Labs: remer tancontian (10), Zairene Lenizo (1). Itisha Joshi (4).
88	Gallinule	720575940633711028	5	56	Murthy and Seung Labs: Claire McKellar (6)
89	SEZ: PRW.PRW.213	720575940625172016	16		Jefferis and Waddell Labs: Joseph Hsu (14). Murthy and Seung Labs: Darrel Jay Akiatan (31), Doug Bland (23), Austin T Burke (1), Ryan Willie (3), remer tancontian (1).
90	SEZ: PRW.GNG.43	720575940630664556	8		Jefferis Lab: Marta Costa (1), Sangeeta Sisodiya (2). Seeds Hampel Lab: Alexis E Santana Cruz (2). Murthy and Seung Labs: Zairene Lenizo (3), Darrel Jay Akiatan (5), Ariel Dagohoy (1), Rey Adrian Candilada (2), Joshua Bañez (1), Nash Hadjerol (6), remer tancontian (1).
91	SEZ: PRW.PRW.312	720575940632055521	7		Murthy and Seung Labs: J. Dolorosa (3), Shirleyjoy Serona (103). Jefferis Lab: Arti Yadav (1).
92	SEZ: PRW.PRW.82	720575940616860758	6		Jefferis Lab: Arti Yadav (1). Murthy and Seung Labs: Joshua Bañez (7), Zairene Lenizo (3), Doug Bland (12), J. Dolorosa (2), remer tancontian (1).
93	SEZ: PRW.PRW.164	720575940622457579	6	43	Jefferis Lab: Arti Yadav (1). Murthy and Seung Labs: Michelle Pantujan (2), Shirleyjoy Serona (3).
				1742	

bioRxiv preprint doi: https://doi.org/10.1101/2023.04.06.535891; this version posted April 8, 2023. The copyright holder for this preprint (which was not certified by peer review) is the author/funder, who has granted bioRxiv a license to display the preprint in perpetuity. It is made available under aCC-BY 4.0 International license.

Table S3: CCAP presynaptic partners

Name	Flywire ID	synapses from BiT2 (7205759406216623 32)	synapses from VESa1 (720575940632951 597)	synapses from CCHa2R-RA (7205759406219420 21)	synapses from CCHa2R-RA (7205759406277659 03)	synapses from Cowboy (720575940611730 674)	Total synapses	Tracing contributions (number of edits)
CCAP	72057594064616 0948	18	8	9	5	0	40	Jefferis Lab: Greg Jefferis (15), Zeba Vohra (17), A. Javier (14), Siqi Fang (9), Varun Sane (6), Dhara Kakadiya (4). Jefferis and Wilson: Laia Serratosa Capdevila (1). Murthy and Seung Labs: Michelle Pantujan (1), Shaina Mae Monungolh (1), Rey Adrian Candilada (2), regine salem (1), Nash Hadjerol (1), Joshua Bañez (1).
CCAP	72057594062114 8993	19	14	5	7	5	50	Murthy and Seung Labs: Austin T Burke (5), Rey Adrian Candilada (1), J. Anthony Ocho (8), Nash Hadjerol (26), Joshua Bañez (4), Ryan Willie (2). Jefferis Lab: A. Javier (28), Imaan Tamimi (1), Katharina Eichler (11), Mendell Lopez (80). Jefferis and Wilson Labs: Laia Serratosa Capdevila (2), Varun Sane (1). Janelia tracers: Tansy Yang (1).
Total synapses per cell type		37	22	14	12	5	90	

bioRxiv preprint doi: https://doi.org/10.1101/2023.04.06.535891; this version posted April 8, 2023. The copyright holder for this preprint (which was not certified by peer review) is the author/funder, who has granted bioRxiv a license to display the preprint in perpetuity. It is made available under aCC-BY 4.0 International license.

Table S4: Fly genotypes in figures

Figure	Short Genotype	Full Genotype
1A	nSyb	w1118 ; UAS-dcr2/+ ; UAS-nSynaptobrevin RNAi (attP2)/+
1A	nSyb	w1118 ; UAS-dcr2/+; UAS-nSynaptobrevin (attP2)/VT011155-Gal4 (attP2)
1A	TRH	w1118 ; UAS-dcr2/+ ; UAS-Trh RNAi (attP2)/+
1A	TRH	w1118 ; UAS-dcr2/+ ; UAS-Trh RNAi (attP2)/VT011155-Gal4 (attP2)
1A	ChAT	w1118 ; UAS-dcr2/+ ; UAS- ChAT RNAi (attP2)/+
1A	ChAT	w1118 ; UAS-dcr2/+ ; UAS- ChAT RNAi (attP2)/VT011155-Gal4 (attP2)
1A	TBH	w1118 ; UAS-dcr2/+ ; UAS-Tbh RNAi (attP2)/+
1A	TBH	w1118 ; UAS-dcr2/+ ; UAS-Tbh RNAi (attP2)/VT011155-Gal4 (attP2)
1A	HDC	w1118 ; UAS-dcr2/+ ; UAS-Hdc RNAi (attP2)/+
1A	HDC	w1118 ; UAS-dcr2/+ ; UAS-Hdc RNAi (attP2)/VT011155-Gal4 (attP2)
1A	VMAT	w1118 ; UAS-dcr2/+; UAS-VMAT RNAi (attP2)/+
1A	VMAT	w1118 ; UAS-dcr2/+ ; UAS-VMAT RNAi (attP2)/VT011155-Gal4 (attP2)
1A	GAD1	w1118 ; UAS-dcr2/+; UAS-GAD1 RNAi (attP2)/+
1A	GAD1	w1118 ; UAS-dcr2/+ ; UAS-GAD1 RNAi (attP2)/VT011155-Gal4 (attP2)
1A	DDC	w1118 ; UAS-dcr2/+ ; UAS-DDC RNAi (attP2)/+
1A	DDC	w1118 ; UAS-dcr2/+ ; UAS-DDC RNAi (attP2)/VT011155-Gal4 (attP2)
1A	DVGlut	w1118 ; UAS-dcr2/+ ; UAS-DVGlut RNAi (attP2)/+
1A	DVGlut	w1118 ; UAS-dcr2/+ ; UAS-DVGlut RNAi (attP2)/VT011155-Gal4 (attP2)
1A	sNPF	w1118 ; UAS-dcr2/+ ; UAS-sNPF RNAi (attP2)/+
1A	sNPF	w1118 ; UAS-dcr2/+ ; UAS-sNPF RNAi (attP2)/VT011155-Gal4 (attP2)
1A	VGAT	w1118 ; UAS-dcr2/+ ; UAS-VGAT RNAi (attP2)/+
1A	VGAT	w1118 ; UAS-dcr2/+ ; UAS-VGAT RNAi (attP2)/VT011155-Gal4 (attP2)
1A	TDC2	w1118 ; UAS-dcr2/+ ; UAS-Tdc2 RNAi (attP2)/+
1A	TDC2	w1118 ; UAS-dcr2/+ ; UAS-Tdc2 RNAi (attP2)/VT011155-Gal4 (attP2)
1A	dILP1	w1118 ; UAS-dcr2/+ ; UAS-dILP1 RNAi (attP2)/+
1A	dILP1	w1118 ; UAS-dcr2/+ ; UAS-dILP1 RNAi (attP2)/VT011155-Gal4 (attP2)
1A	dILP2	w1118 ; UAS-dcr2/+ ; UAS-dILP2 RNAi (attP2)/+
1A	dILP2	w1118 ; UAS-dcr2/+ ; UAS-dILP2 RNAi (attP2)/VT011155-Gal4 (attP2)
1A	dILP3	w1118 ; UAS-dcr2/+ ; UAS-dILP3 RNAi (attP2)/+

bioRxiv preprint doi: https://doi.org/10.1101/2023.04.06.535891; this version posted April 8, 2023. The copyright holder for this preprint (which was not certified by peer review) is the author/funder, who has granted bioRxiv a license to display the preprint in perpetuity. It is made available under aCC-BY 4.0 International license.

1A	dILP3	w1118 ; UAS-dcr2/+ ; UAS-dILP3 RNAi (attP2)/VT011155-Gal4 (attP2)
1A	dILP4	w1118 ; UAS-dcr2/+ ; UAS-dILP4 RNAi (attP2)/+
1A	dILP4	w1118 ; UAS-dcr2/+ ; UAS-dILP4 RNAi (attP2)/VT011155-Gal4 (attP2)
1A	dILP5	w1118 ; UAS-dcr2/+ ; UAS-dILP5 RNAi (attP2)/+
1A	dILP5	w1118 ; UAS-dcr2/+ ; UAS-dILP5 RNAi (attP2)/VT011155-Gal4 (attP2)
1A	dILP6	w1118 ; UAS-dcr2/+ ; UAS-dILP6 RNAi (attP2)/+
1A	dILP6	w1118 ; UAS-dcr2/+ ; UAS-dILP6 RNAi (attP2)/VT011155-Gal4 (attP2)
1A	dILP7	w1118 ; UAS-dcr2/+ ; UAS-dILP7 RNAi (attP2)/+
1A	dILP7	w1118 ; UAS-dcr2/+ ; UAS-dILP7 RNAi (attP2)/VT011155-Gal4 (attP2)
1B	dILP3 RNAi	w1118 ; + ; UAS-dILP3 RNAi (attP2)/+
1B	dILP3 RNAi	w1118 ; + ; UAS-dILP3 RNAi (attP2)/VT011155-Gal4 (attP2)
1B	Amon RNAi	w1118/w* ; + ; UAS-amon RNAi (attP2)/+
1B	Amon RNAi	w1118/w* ; + ; UAS-amon RNAi (attP2)/VT011155-Gal4 (attP2)
1B	ISN-Gal4	w1118 ; + ; VT011155-Gal4 (attP2)/+
1D	ISN-Gal4 > tdT	w1118/w* ; UAS-myrGFP.QUAS-mtdTomato-3xHA/+; trans-Tango/VT011155-Gal4 (attP2)
2B,2F	BiT split-Gal4 > Chrimson	w1118, UAS-IVS-CsChrimson.mVenus (attP18)/+; VT002073-Gal4.AD (attP40)/+; VT040568-Gal4.DBD (attP2)/+
2D, 2E	ISN > Chrimson, BiT > ArcLight	13XLexAop2-IVS-p10-ChrimsonR-mCherry (attP18)/w1118; GMR34G02-LexA (attP40)/VT002073-Gal4.AD (attP40); VT040568-Gal4.DBD (attP2)/UAS-ArcLight (attP2)
2F, 2G	BiT split-Gal4	w1118; VT002073-Gal4.AD (attP40)/+; VT040568-Gal4.DBD (attP2)/+
2F	Empty split-Gal4 > Chrimson	w1118, UAS-IVS-CsChrimson.mVenus (attP18); p65-AD.empty (attp40)/+; GAL4-DBD.empty (attP2)/+
2F	Gr5a-Gal4 > Chrimson	w1118, UAS-IVS-CsChrimson.mVenus (attP18); Gr5a-Gal4/+; Gr5a-Gal4/+
2F	ppk28-Gal4 > Chrimson	w1118, UAS-IVS-CsChrimson.mVenus (attP18); + ; ppk28-Gal4/+
2G	BiT split-Gal4 > nSyb RNAi	w1118; VT002073-Gal4.AD (attP40)/+; VT040568-Gal4.DBD (attP2)/UAS-nSynaptobrevin RNAi (attP2)
2G, 4G, 5G	nSyb RNAi	w1118; +; UAS-nSynaptobrevin RNAi (attP2)/+
4B, 4F	CCHa2R (RA) > Chrimson	w1118, UAS-IVS-CsChrimson.mVenus (attP18)/w1118; TI{2A-GAL4}CCHa2-R[2A-A.GAL4]/+; +
4D, 4E	ISN > Chrimson, CCHa2R (RA) > GCaMP	w1118; GMR34G02-LexA (attP40)/ TI{2A-GAL4}CCHa2-R[2A-A.GAL4]; LexAop-Chrimson,UAS-GCaMP6s/TM2
4F, 4G	CCHa2R (RA)	w1118; TI{2A-GAL4}CCHa2-R[2A-A.GAL4]/+; +
4F, 5F	UAS-Chrimson	w1118, UAS-IVS-CsChrimson.mVenus (attP18)/w1118; +; +

bioRxiv preprint doi: https://doi.org/10.1101/2023.04.06.535891; this version posted April 8, 2023. The copyright holder for this preprint (which was not certified by peer review) is the author/funder, who has granted bioRxiv a license to display the preprint in perpetuity. It is made available under aCC-BY 4.0 International license.

4G	CCHa2R (RA) > nSyb RNAi	w1118; TI{2A-GAL4}CCHa2-R[2A-A.GAL4]/+; UAS-nSynaptobrevin RNAi (attP2)/+
5B, 5F	CCAP > Chrimson	w1118, UAS-IVS-CsChrimson.mVenus (attP18)/w1118; CCAP-Gal4/+; +
5D, 5E	ISN > Chrimson, CCAP > GCaMP	w1118, 13XLexAop2-IVS-p10-ChrimsonR-mCherry (attP18)/w1118; GMR34G02-LexA (attP40)/CCAP-Gal4; 20XUAS-IVS-jGCaMP7b(VK00005)/+
5F, 5G	CCAP	w1118; CCAP-Gal4/+; +
5G	CCAP > NSyb RNAi	w1118; CCAP-Gal4/+; UAS-nSynaptobrevin RNAi (attP2)/+
Supp 1A	ISN > transTango	w*/w1118 ; UAS-myrGFP.QUAS-mtdTomato-3xHA/+; trans-Tango/VT011155-Gal4 (attP2)
Supp 2A,B	BiT genetic control	w1118, 13XLexAop2-IVS-p10-ChrimsonR-mCherry (attP18)/w1118; VT002073-Gal4.AD (attP40)/+; VT040568-Gal4.DBD (attP2)/UAS-ArcLight (attP2)
Supp 3A,B	BiT > Chrimson, IPC > GCaMP	10XUAS-syn21-Chrimson88-tdT3.1 (attP18), LexAop2-syn21-opGCaMP6s suHw (attP8)/w1118; TI{2A-lexA::GAD}CCHa2-R[2A-A.lexA]/VT002073-Gal4.AD (attP40); VT040568-Gal4.DBD (attP2)/+
Supp 3C,D	IPC genetic control	10XUAS-syn21-Chrimson88-tdT3.1 (attP18), LexAop2-syn21-opGCaMP6s suHw (attP8)/w1118; dILP2-LexA/CyO; TM2/TM3, Sb
Supp 4A,B	CCHa2R (RA) genetic control	w1118; TI{2A-GAL4}CCHa2-R[2A-A.GAL4]/CyO; LexAop-Chrimson,UAS-GCaMP6s/TM2
Supp 4C,D	BiT > Chrimson, CCHa2R (RA) > GCaMP	10XUAS-syn21-Chrimson88-tdT3.1 (attP18), LexAop2-syn21-opGCaMP6s suHw (attP8)/w1118; TI{2A-lexA::GAD}CCHa2-R[2A-A.lexA]/VT002073-Gal4.AD (attP40); VT040568-Gal4.DBD (attP2)/+
Supp 5	ISN > Chrimson, CCAP > GCaMP	13XLexAop2-IVS-p10-ChrimsonR-mCherry (attP18)/w1118; GMR34G02-LexA (attP40)/+ ; 20XUAS-IVS-jGCaMP7b(VK00005)/CCAP-Gal4

bioRxiv preprint doi: https://doi.org/10.1101/2023.04.06.535891; this version posted April [illegible]. The copyright holder for this preprint (which was not certified by peer review) is the author/funder, who has granted bioRxiv a license to display the preprint in perpetuity. It is made available under aCC-BY 4.0 International license.

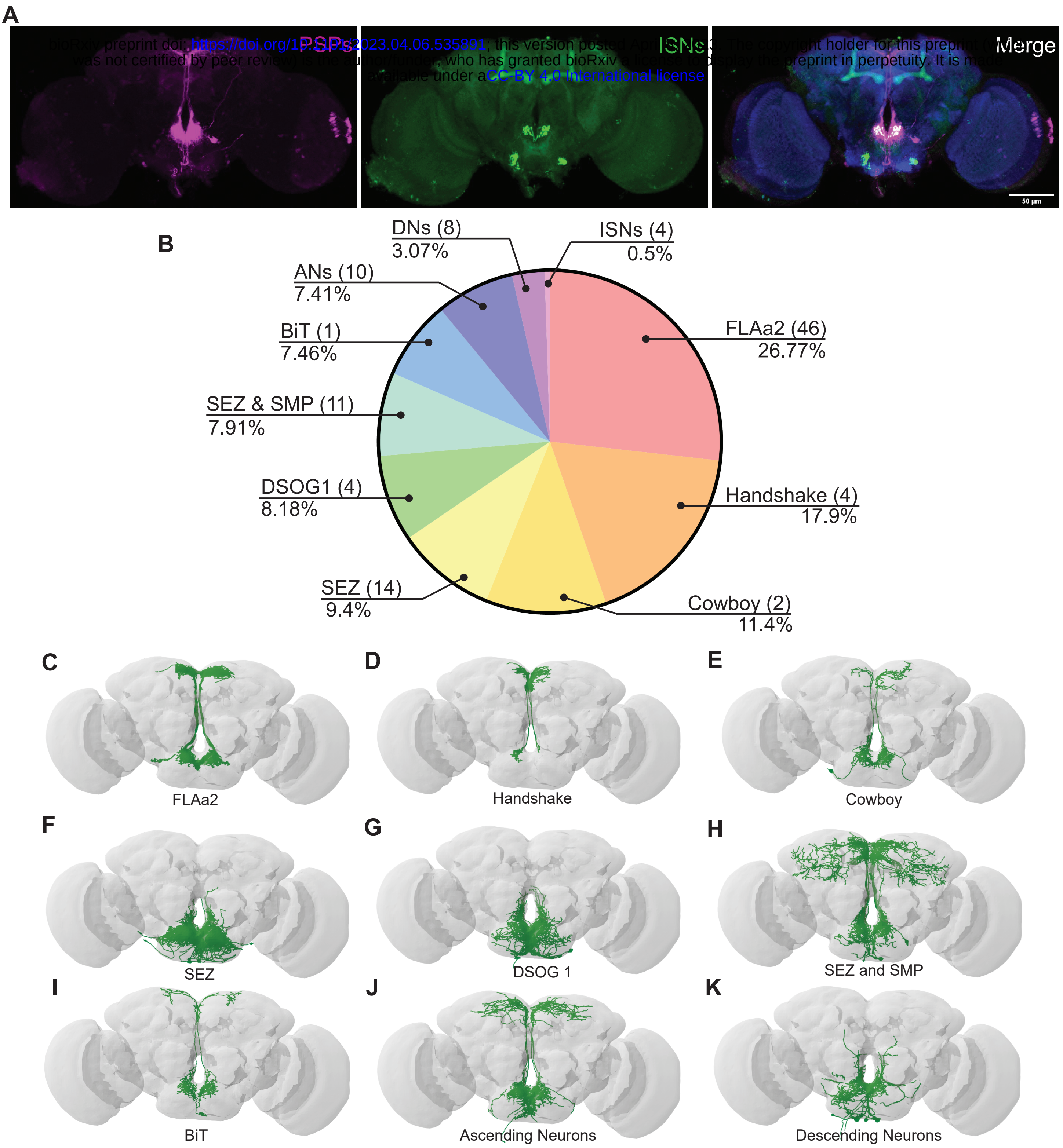

Figure S1. ISN postsynaptic partners labeled by trans-Tango and EM, Related to Figure 1
(A) Expression of trans-Tango ligand in the ISNs (green) and postsynaptic partners (PSPs) (magenta). nc82 staining in blue. (B) Distribution of synaptic output from the ISNs divided by cell class or brain region. Total of 4050 synapses from the ISNs and 104 postsynaptic partners. FLAa2 (46 neurons) receive 26.77% of all ISN output, Handshake (4 neurons) 17.9%, Cowboy (2 neurons) 11.4%, neurons located in the subesophageal zone (SEZ) (14 neurons) 9.04%, DSOG1 (4 neurons) 8.18%, neurons with neurites in the subesophageal zone and superior medial protocerebrum (SEZ & SMP) (11 neurons) 7.91%, BiT (1 neuron) 7.46%, Ascending neurons (ANs) (10 neurons) 4.41%, Descending neurons (DNs) (8 neurons) 3.07%, and ISNs (4 neurons) 0.5%. Only postsynaptic partners with 5 or more synapses were considered for this analysis. Reconstruction of FLAa2 neurons (C), Handshake neurons (D), Cowboy neurons (E), neurons innervating the SEZ (F), DSOG1 neurons (G), neurons innervating the SEZ & SMP (H), BiT (I), ascending neurons (J), descending neurons (K).

bioRxiv preprint doi: https://doi.org/10.1101/2023.04.06.535891; this version posted April 8, 2023. The copyright holder for this preprint (which was not certified by peer review) is the author/funder, who has granted bioRxiv a license to display the preprint in perpetuity. It is made available under aCC-BY 4.0 International license.

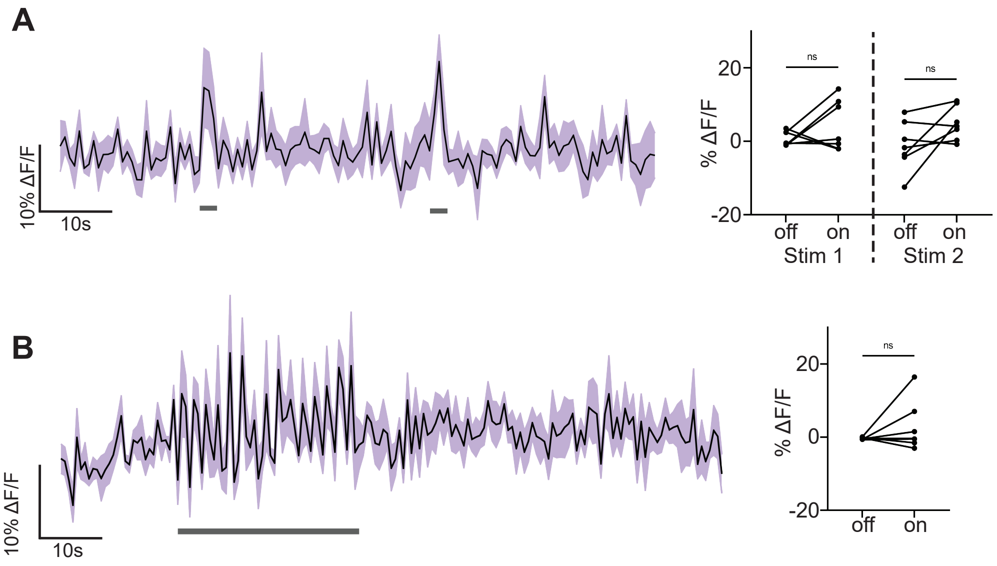

Figure S2. BiT genetic control functional imaging, Related to Fig 2
Genetic controls: we expressed the light sensitive ion channel Chrimson without the ISN-LexA driver and exposed the brains to 660nm LED. We expressed the voltage sensor ArcLight in BiT and imaged it with a 2 photon microscope. (A) ArcLight response of BiT soma to 2s LED exposure or (B) 30s LED exposure. Left: Scatter plot shows mean +/- SEM of all flies imaged, gray bars represent LED stimulation. Right: Quantification of mean fluorescence intensity before stim (off) and during stim (on), each dot represents one fly. Paired Wilcoxon or paired t-test. n=7 flies.

bioRxiv preprint doi: https://doi.org/10.1101/2023.04.06.535891; this version posted April 8, 2023. The copyright holder for this preprint (which was not certified by peer review) is the author/funder, who has granted bioRxiv a license to display the preprint in perpetuity. It is made available under aCC-BY 4.0 International license.

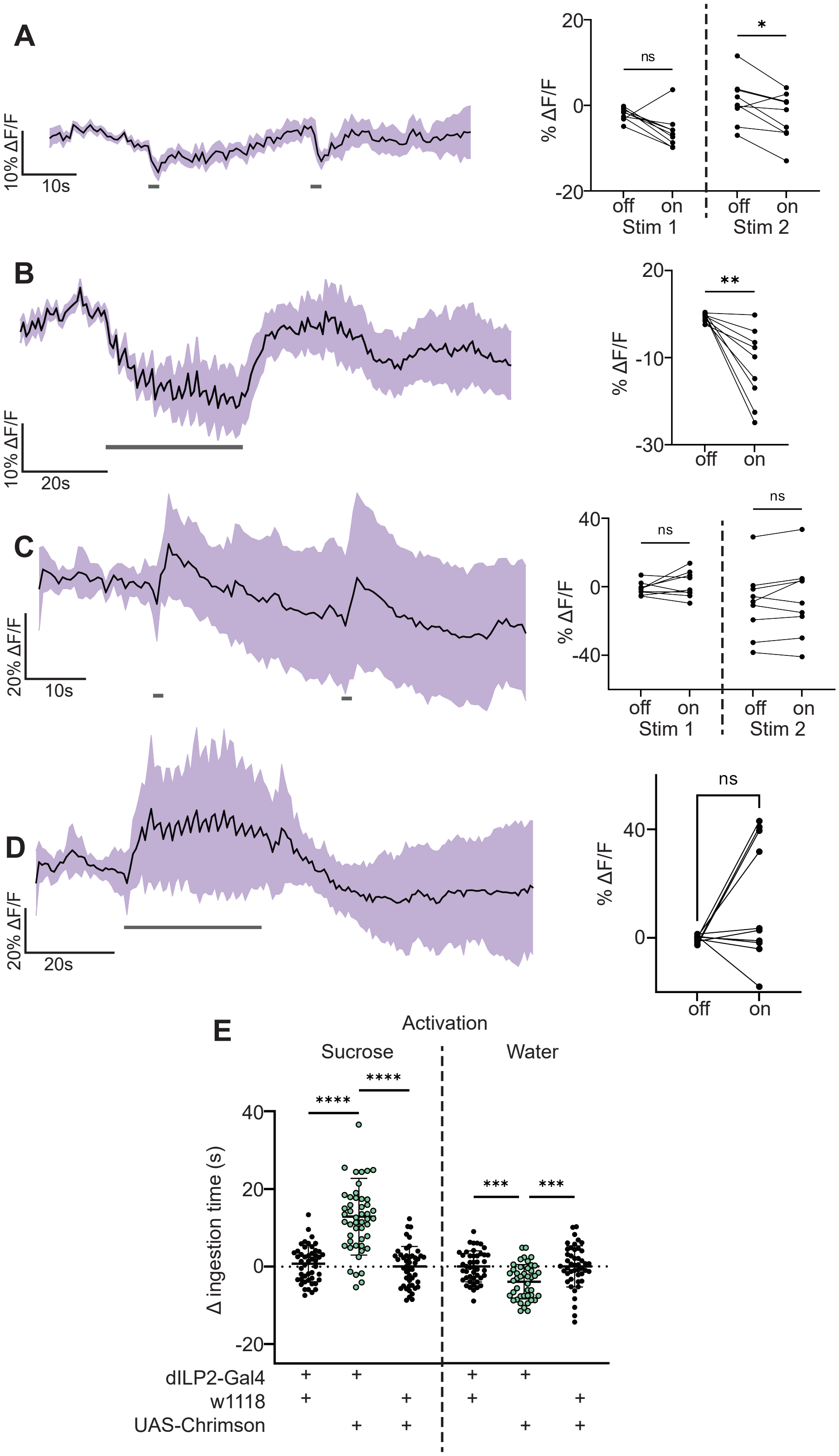

Figure S3. IPC response to BiT stimulation, Related to Fig 3
A and B: We expressed the light sensitive ion channel Chrimson in BiT and optogenetically stimulated it with 660nm LED. We expressed the calcium sensor GCaMP in the IPCs and imaged them with a 2 photon microscope. (A) Calcium response of IPC somas to 2s optogenetic activation of BiT or (B) 30s optogenetic activation of BiT. Left: Scatter plot shows mean +/- SEM of all flies imaged, gray bars represent LED stimulation. Right: Quantification of mean fluorescence intensity before stim (off) and during stim (on), each dot represents one fly. Paired Wilcoxon test or paired t-test. n=9 flies. C and D: Genetic controls: we expressed the light sensitive ion channel Chrimson without the BiT-split Gal4 driver and exposed the brains to 660nm LED. We expressed the calcium sensor GCaMP in the IPCs and imaged them with a 2 photon microscope. (C) Calcium response of IPC somas to 2s LED exposure or (D) 30s LED exposure. Left: Scatter plot shows mean +/- SEM of all flies imaged, gray bars represent LED stimulation. Right: Quantification of mean fluorescence intensity before stim (off) and during stim (on), each dot represents one fly. Paired t-test. n=9-10 flies. (E) Temporal consumption assay for 1M sucrose or water during acute optogenetic activation of IPCs with Chrimson. Ingestion time of females exposed to light normalized to dark controls of indicated genotype. One-way ANOVA with Holm-Šídák multiple comparison test. n=43-49 animals/genotype. *p<0.05, **p<0.01, ***p<0.001, ****p<0.0001

bioRxiv preprint doi: https://doi.org/10.1101/2023.04.06.535891; this version posted April 8, 2023. The copyright holder for this preprint (which was not certified by peer review) is the author/funder, who has granted bioRxiv a license to display the preprint in perpetuity. It is made available under aCC-BY 4.0 International license.

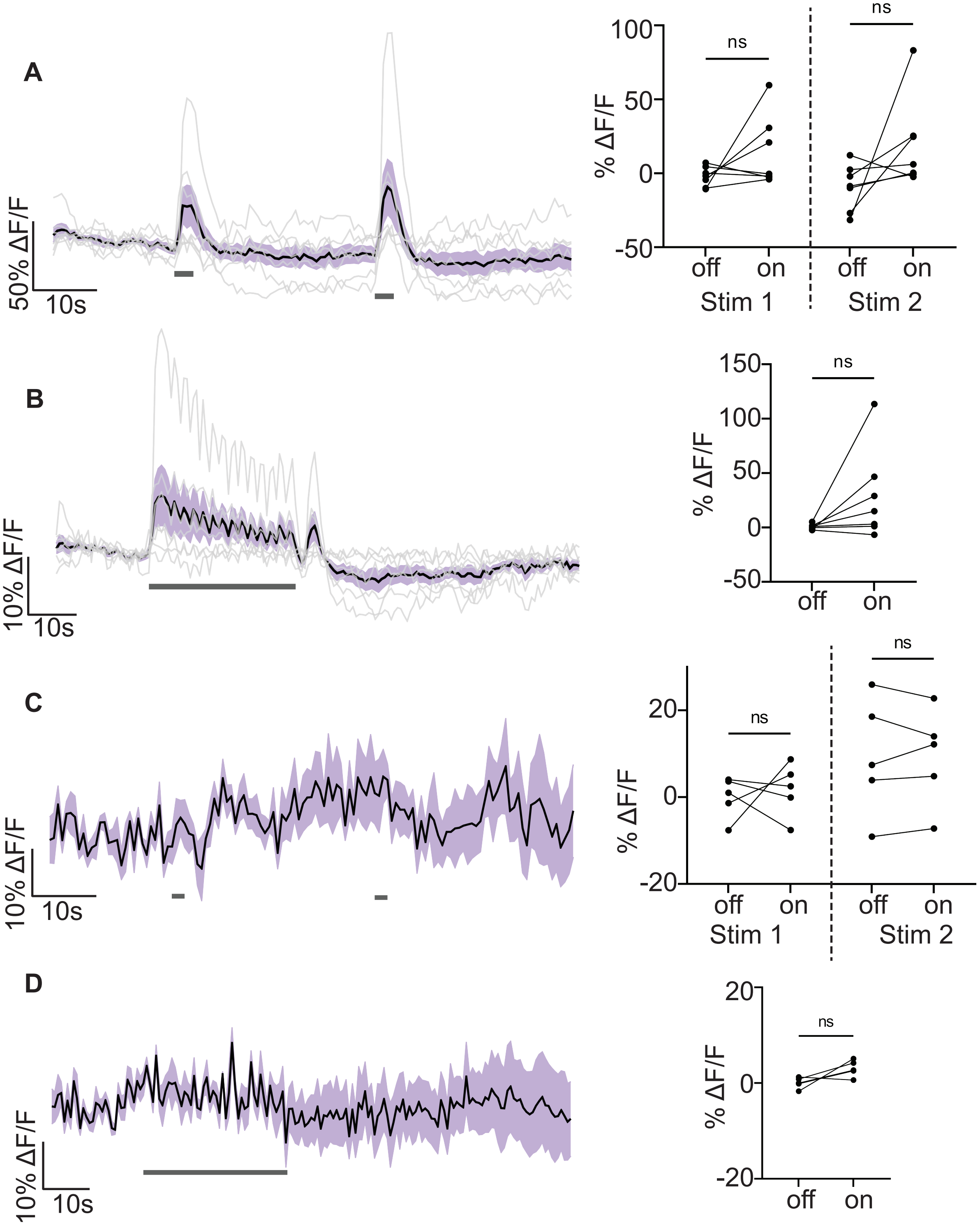

Figure S4. CCHa2R-RA genetic controls functional imaging and response to BiT optogenetic stimulation, Related to Fig 4

A and B: Genetic controls: we expressed the light sensitive ion channel Chrimson without the ISN-LexA driver and exposed the brains to 660nm LED. We expressed the calcium sensor GCaMP in the CCHa2R-RA neurons and imaged them with a 2 photon microscope. (A) Calcium response of CCHa2R-RA SEZ neurites to 2s LED exposure or (B) 30s LED exposure. Left: Scatter plot shows mean +/- SEM of all flies imaged, with individual traces in gray, gray bars represent LED stimulation. Right: Quantification of mean fluorescence intensity before stim (off) and during stim (on), each dot represents one fly. Paired Wilcoxon test and paired t-test. n=7 flies. C and D: We expressed the light sensitive ion channel Chrimson in BiT and optogenetically stimulated it with 660nm LED. We expressed the calcium sensor GCaMP in the CCHa2R-RA neurons and imaged them with a 2 photon microscope. (C) Calcium response of CCHa2R-RA somas to 2s optogenetic stimulation of BiT or (D) 30s optogenetic stimulation of BiT. Left: Scatter plot shows mean +/- SEM of all flies imaged, gray bars represent LED stimulation. Right: Quantification of mean fluorescence intensity before stim (off) and during stim (on), each dot represents one fly. Paired t-test. n=5 flies.

bioRxiv preprint doi: https://doi.org/10.1101/2023.04.06.535891; this version posted April 8, 2023. The copyright holder for this preprint (which was not certified by peer review) is the author/funder, who has granted bioRxiv a license to display the preprint in perpetuity. It is made available under aCC-BY 4.0 International license.

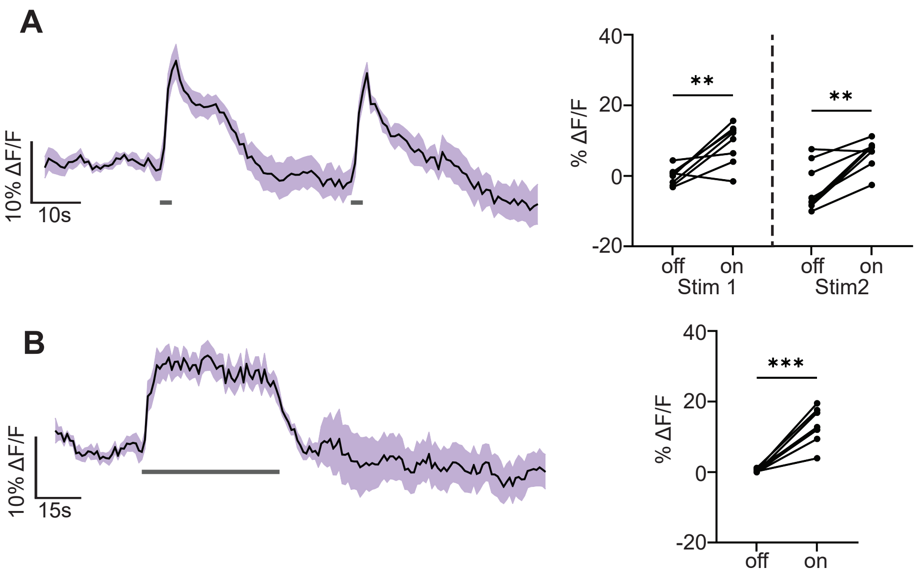

Figure S5. CCAP respond to ISN activation using another CCAP-Gal4 driver, Related to Fig 5

We expressed the light sensitive ion channel Chrimson in the ISNs and optogenetically stimulated them with 660nm LED. We expressed the calcium sensor GCaMP in the CCAP neurons and imaged them with a 2 photon microscope. (A) Calcium response of CCAP neurites to 2s optogenetic stimulation of the ISNs or (B) 30s optogenetic stimulation of the ISNs. Left: Scatter plot shows mean +/- SEM of all flies imaged, gray bars represent LED stimulation. Right: Quantification of mean fluorescence intensity before stim (off) and during stim (on), each dot represents one fly. Paired t-test. n=8 flies. **p<0.01, ***p<0.001
